# Application of 2,4-Dinitrophenylhydrazine (DNPH) in High-Throughput Screening for Microorganism Mutants Accumulating 9α-Hydroxyandrost-4-ene-3,17-dione (9α-OH-AD)

**DOI:** 10.1371/journal.pone.0163836

**Published:** 2016-10-05

**Authors:** Yang Liu, Fei Cao, Hui Xiong, Yanbing Shen, Min Wang

**Affiliations:** Key Laboratory of Industrial Fermentation Microbiology, Ministry of Education, College of Biotechnology, Tianjin University of Science & Technology, Tianjin, People's Republic of China; National Renewable Energy Laboratory, UNITED STATES

## Abstract

To develop a quick method for the preliminarily screening of mutant strains that can accumulate 9α-hydroxyandrost-4-ene-3,17-dione (9α-OH-AD), a high-throughput screening method was presented by applying the principle that 2,4-dinitrophenylhydrazine (DNPH) can react with ketones to produce precipitation. The optimal color assay conditions were the substrate androst-4-ene-3,17-dione (AD) concentration at 2.0 g/L, the ratio of AD to DNPH solution at 1:4, and the sulfuric acid and ethanol solution percentages in DNPH solution at 2% and 35%, respectively. This method was used to preliminarily screen the mutants of *Rhodococcus rhodochrous* DSM43269, from which the three ones obtained could produce more 9α-OH-AD. This DNPH color assay method not only broadens screening methods and increases screening efficiency in microbial mutation breeding but also establishes a good foundation for obtaining strains for industrial application.

## Introduction

9α-Hydroxysteroids, which contain a hydroxyl group in the C-9α position of their steroid polyheterocyclic molecules, can be used as starting compounds for the synthesis of 9α-halo-11β-hydroxysteroids, such as 9α-fluorohydrocortisone, triamcinolone, dexamethasone and synaflan that possess high antiallergic, antishock and anti-inflammatory activities [1, 2]. Chemical processes and microbial transformations can be employed to produce 9α-hydroxysteroids. It is difficult to introduce hydroxyl groups into the C-9α position of the steroid molecules by organic synthesis. On the contrary, microbial transformations, which are enzymatic reactions catalyzed by microorganisms, can easily exert reactions of stereo- and region- selectivity, and have many other advantages over chemical processes, e.g., mild reaction conditions and lower costs.

9α-Hydroxyandrost-4-ene-3,17-dione (9α-OH-AD), one of the important 9α-hydroxysteroids, can be obtained from the hydroxylation of androst-4-ene-3,17-dione (AD) by microorganisms. The microbial transformation to 9α-OH-AD mainly focuses on some mycolic acid-containing Actinomycete, such as *Rhodococcus* [2–4], *Mycobacterium* [5, 6], and *Nocardia* [7]. However, these mycolic acid-containing bacteria can utilize steroids as their sole carbon and completely degrade AD or 9α-OH-AD into CO_2_ and H_2_O. This is due to the introduction of 9α-hydroxyl moiety into the steroid polyheterocyclic ring structure which is caused by 3-ketosteroid-9α-hydroxylase (KSH), combined with steroid-Δ^1^-dehydrogenation by 3-ketosteroid-Δ^1^-dehydrogenase (KSDD); this process leads to the formation of the chemically unstable 9α-hydroxy-1,4-androstadiene-3,17-dione, which in turn initiates the steroid B-ring opening. To obtain one strain that stably accumulates 9α-OH-AD, gene deletion or mutation breeding is a potent method that can result in KSDD activity deficiency [8–10]. A quick and efficient screening method is required in the application of mutation breeding for the preliminarily selection of mutants to reduce screening work period. To preliminarily screen *Rhodococcus erythropolis* mutants that could stably accumulate 9α-OH-AD, Van der Geize et al. [9] used mineral medium plates with different steroids as sole carbon to screen the mutants which is based on the principle that KSDD deficient mutants can grow normally on mineral medium plates with androst-1,4-diene-3,17-dione (ADD), but not on the plates with AD or 9α-OH-AD. Liu et al. [10] selected *Mycobacterium neoaurum* mutants blocked in KSDD by using a color assay screening method based on the reduced flavin adenine dinucleotide (FADH_2_) of KSDDs that can reduce 2,6-dichlorophenolindophenol (DCPIP). However, these methods are discovered unsuitable for *Rhodococcus rhodochrous* DSM43269 during its mutagenesis work. Considering the particularity of this strain and the possibility of other similar strains existing, our attention turned to the exploration of a suitable and speedy approach to screen mutants.

2,4-Dinitrophenylhydrazine (DNPH) can react with ketones to produce color phenylhydrazone precipitation. If strains are treated with mutation, the KSDD deficient mutants will accumulate 9α-OH-AD that can react with DNPH to produce red precipitation; otherwise, the red precipitation will not form. Thus, combined with the cultivation mode of 24-deep-well plates, a novel color assay was established according to this principle of screening mutants that can accumulate 9α-OH-AD.

## Materials and Methods

### Bacterial strains, culture media, and chemicals

*R*. *rhodochrous* DSM43269 was purchased from China General Microbiological Culture Collection Center. The seed medium (g/L) contained peptone 4, yeast extract 12, glucose 12, and pH 7.0, and the ingredients of the fermentation medium (g/L) consisted of glucose 4, peptone 2, corn steep liquor 24, K_2_HPO_4_ 0.2, and MgSO_4_ 0.1, with a pH of 7.0. AD and 9α-OH-AD were obtained from Sigma Aldrich Co. DNPH was purchased from Sinopharm Chemical Reagent Beijing Co., Ltd (China). The 24-deep-well plates with cover were obtained from Changzhou Yingde Bio-technology Co., Ltd. (China).

### Screening methods

Plate screening method was implemented following the method by Van der Geize et al. [9], except for the liquid medium used in this experiment because the impurity in agar might influence the results of the strain’s growth. The color assay with DCPIP was performed according to the method by Liu et al. [10].

The color assay with DNPH was carried out as follows: *R*. *rhodochrous* DSM43269 was initially incubated in a 250 mL shake flask with 20 mL of the seed medium at 200 rpm and 30°C for about 20 h (OD_600_ 2.0±0.1). Afterward, 1 mL of culture was transferred into a new 250 mL shake flask with 20 mL of the seed medium to continue growth for approximately 20 h under the same condition. The substrate AD dissolved in methanol was added into the culture at the final concentration of 2.0 g/L. The transformation was monitored by thin layer chromatography (TLC), and 300 μL of the culture was extracted and fully mixed with 300 μL of ethyl acetate to obtain the organic solutions. Afterward, 0.4 g DNPH was dissolved in 3.0 mL of concentrated sulfuric acid, slowly poured into 30 mL of ethanol solution (95% ethanol), and diluted with deionized water to form 100 mL of the color assay DNPH solution. Each 100 μL of the color assay solution was added into each well of 96-well plates, followed by 25 μL of the above organic solutions. The reactions were performed at room temperature until red precipitation formed.

### Factors affecting the color assay with DNPH

Substrate AD was initially dissolved in ethyl acetate to obtain AD solutions of final concentration at 0, 0.5, 1.0, 1.5, 2.0, 2.5, 3.0, 3.5, 4.0, 4.5, and 5.0 g/L. These AD solutions reacted with the above DNPH color assay solution. Afterward, the total volume of the reaction was kept unchanged, and the volume ratio of AD to DNPH solution (1:1–1:5) was altered. The percentage of sulfuric acid (1%–5%, v/v) or ethanol solution (20%–40%, v/v) in the DNPH solution was then altered, and the resulting DNPH solutions were used to individually react with AD solution. Each reaction was carried out for 5 min.

After the reactions, the 96-well plates were photographed to images under the same light condition. Photoshop CS6 was used to transform the resulting images to gray ones, reference line was added to divide each well of the plates into two equal parts, and three spots on the reference line were selected to read their gray values, which were in the homogeneous precipitation area in the wells. Gray values were used to quantify the color depth which was proportional to the extent of reaction.

### Cultivation mode in 24-deep-well plates

*R*. *rhodochrous* DSM43269 was pre-cultured in a 500 mL shake flask with 100 mL of the seed medium at 200 rpm and 30°C for about 24 h. The resulting culture was then divided into the 24-deep-well plates and 250 mL shake flasks individually to continue growth under the same condition. The liquid volumes in 24-deep-well plates were set at 1.1, 1.4, 1.7, 2.0, and 2.3 mL to compare their cell growth and evaporation rate with those of the culture at 20 mL in the shake flask every 24 h. All experiments were carried out in triplicate. The optical density of 600 nm wavelength was used to monitor the cell growth. The volume of the culture at 0 h was set as V_0_ (mL), and the volume of the culture every 24 h was represented as Vt (mL). Thus, the evaporation rate (E) was calculated using the following function:

E(%)=(1−VtV0)×100% (1)

### Mutagenesis treatment of the strain

The Atmospheric and Room Temperature Plasma (ARTP) biological breeding system was used to mutate the wild-type *R*. *rhodochrous* DSM43269 in this study. The original strain was cultivated in the seed medium at 30°C and 200 rpm for 48 h. The cells were collected, washed twice, and re-suspended with 0.9% of sterilized saline solution and then adjusted to 2 × 10^8^ cfu/mL concentration. A 10 μL aliquot of the culture solution was applied to a sterilized stainless steel plate and treated by the helium plasma jet for a particular time. The distance between the plasma torch nozzle exit and the sample plate was adjusted to 2 mm. The apparatus was operated at radio frequency power input of 40 W and helium gas flow of 10 L/min. The plasma jet temperature was below 40°C, and the plasma treatment time ranged from 0 s to 80 s. After the sample treatment, the sample plates were placed into new tubes and washed with 2 mL of sterilized saline solution. Afterward, 100 μL of the culture solution was spread on the solid seed medium plate and cultivated at 30°C for about 4 days.

### Steroid transformation and analysis

The cultivation and substrate transformation in the fermentation medium of the wild-type *R*. *rhodochrous* DSM43269 and its mutants obtained from mutagenesis were performed in line with the seed medium approach in the shake flask above. Each conversion was done in triplicate and sampled every 3 h, and 300 μL of the culture was extracted and fully mixed with 600 μL of ethyl acetate. The mixture was centrifugated for 1 min at 12000×g, and 100 mL of the organic phase was transferred into a clean tube. The steroid extract was dried at room temperature, re-dissolved in 1 mL of 80% methanol, and filtered through 0.22 μm of the microporous membrane. The HPLC-UV_254_ with an Agilent XDB-C18 column (250×4.6 mm; 5 μm) was performed at 35°C, using methanol-water (80:20, v/v) as the mobile phase at a 1 mL/min flow rate.

## Results

### Screening method establishment

**Fig 1:**
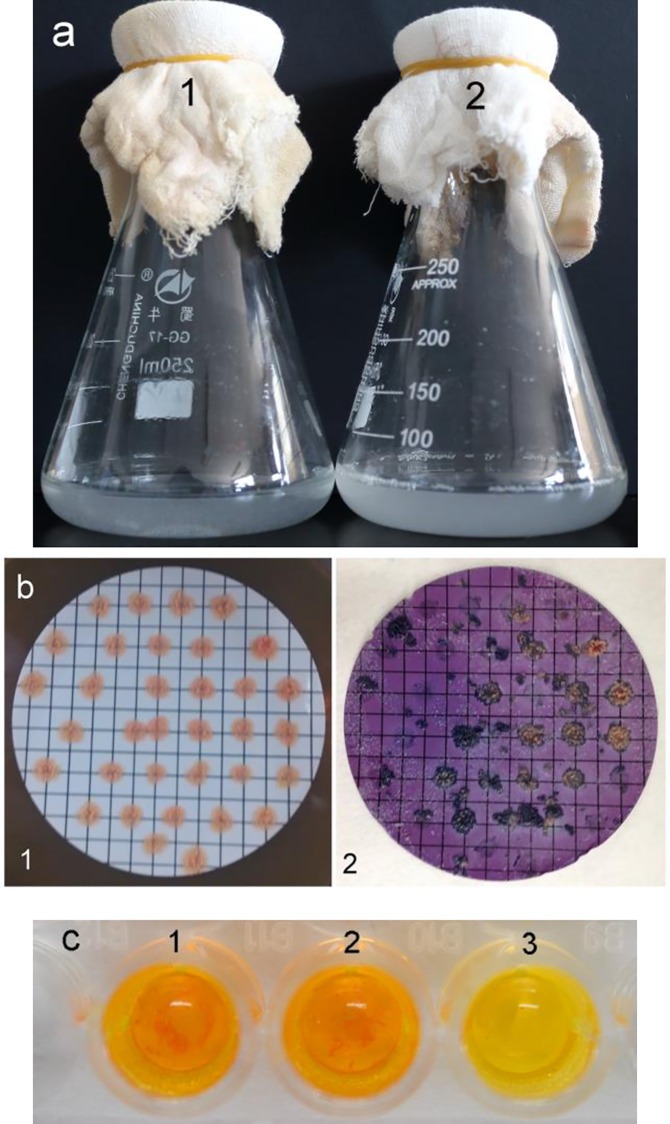
Various high-throughput screening methods. (a) Plate screening method where 1 and 2 indicate the control medium and bacteria growing on medium with 0.5 g/L of AD, respectively. (b) Color assay with DCPIP where 1 and 2 indicate the colonies growing on the gridded sterile membrane filters and colonies incubated with AD, respectively. (c) Color assay with DNPH where 1, 2, and 3 denote the AD standard as well as the product obtained during the process and at the end of the substrate transformation, respectively.

*R*. *rhodochrous* DSM43269, the wild type, was the model used to select screening methods. The strain was initially cultivated in minimal medium with AD as the sole carbon source. Its growth status was continuously observed for 10 days, and the cultivation was stopped until the cultures were unchanged. The medium in which the bacteria grew extremely bad did not show any significant difference from the control minimal medium (Fig 1A). This finding may be because the *R*. *rhodochrous* DSM43269 cannot grow well unless sufficient carbon sources are present in the medium. Afterward, the color assay with DCPIP was carried out. Fig 1B shows the good growth of the strain on the filters as each membrane was filled with colonies. Most of the colonies shift or fell off from the filters, losing many available colonies and displaying unobvious color change, after dyeing with DCPIP for 24 h. Thus, these results confirm the unsuitability of these methods for *R*. *rhodochrous* DSM43269.

The culture liquid of the strain was extracted with ethyl acetate during the process and at the end of the substrate conversion. The product, which was obtained during the transformation process, reacted with DNPH to form red precipitation, which suggests the existence of compounds that contained keto group (Fig 1C). Furthermore, the product gained from the end of the transformation failed to form red precipitation. Their TLC assays exhibited the consistent results, demonstrating that DNPH was competent in analyzing the product accumulation of *R*. *rhodochrous* DSM43269.

### Optimal conditions of the color assay

**Fig 2:**
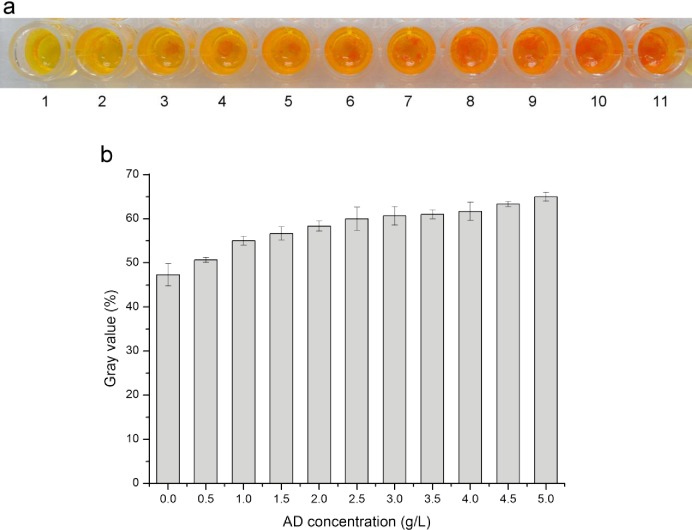
Color assay of the DNPH reaction with AD of different concentrations. (a) Color assay comparison. The AD concentration from left to right is 0, 0.5, 1.0, 1.5, 2.0, 2.5, 3.0, 3.5, 4.0, 4.5, and 5.0 g l^-1^. (b) Gray value of the color assay results. Values are presented as means ± SD (n = 3).

The results of the DNPH reaction with substrate AD of different concentrations are displayed in Fig 2A, and their corresponding amounts are presented in gray value (Fig 2B). When AD concentration was 0.5 g/L, very weak red precipitation formed. As the AD concentration rose, the red precipitation amount increased and became increasingly apparent as well. Considering the above work, the wild type *R*. *rhodochrous* DSM43269 can completely decompose 2.0 g/L of AD in no more than 48 h. Moreover, 2.0 g/L of AD can produce distinct red precipitation with DNPH, as shown in Fig 2. Thus, the optimal substrate concentration to screen mutants in a short time was set as 2.0 g/L.

**Fig 3:**
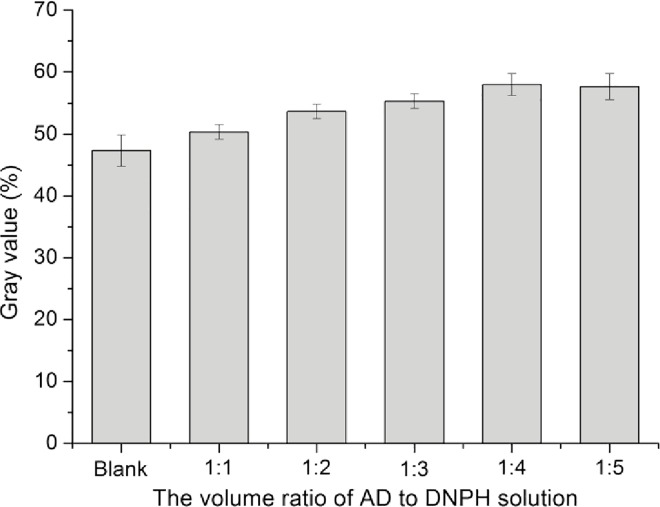
Ratio of AD to DNPH solution on the color assay effect. Values are presented as means ± SD (n = 3).

This method aimed to use vision as the basis for judging so that the compound generated from the DNPH and ketones reaction is precipitation, which cannot be quantified by light absorption value. Moreover, the gray value can express the color difference of various substrate concentrations well. Therefore, the following experiments were represented in their corresponding gray values. Based on the 2.0 g/L AD solution, the effect of the AD to DNPH solution volume ratio on the color assay was investigated, and as a result (Fig 3), the red precipitation became increasingly evident as the AD to DNPH solution volume ratio decreased. The color was most obvious when the ratio was 1:4. Thus, the optimal ratio of AD to DNPH solution was 1:4.

**Fig 4:**
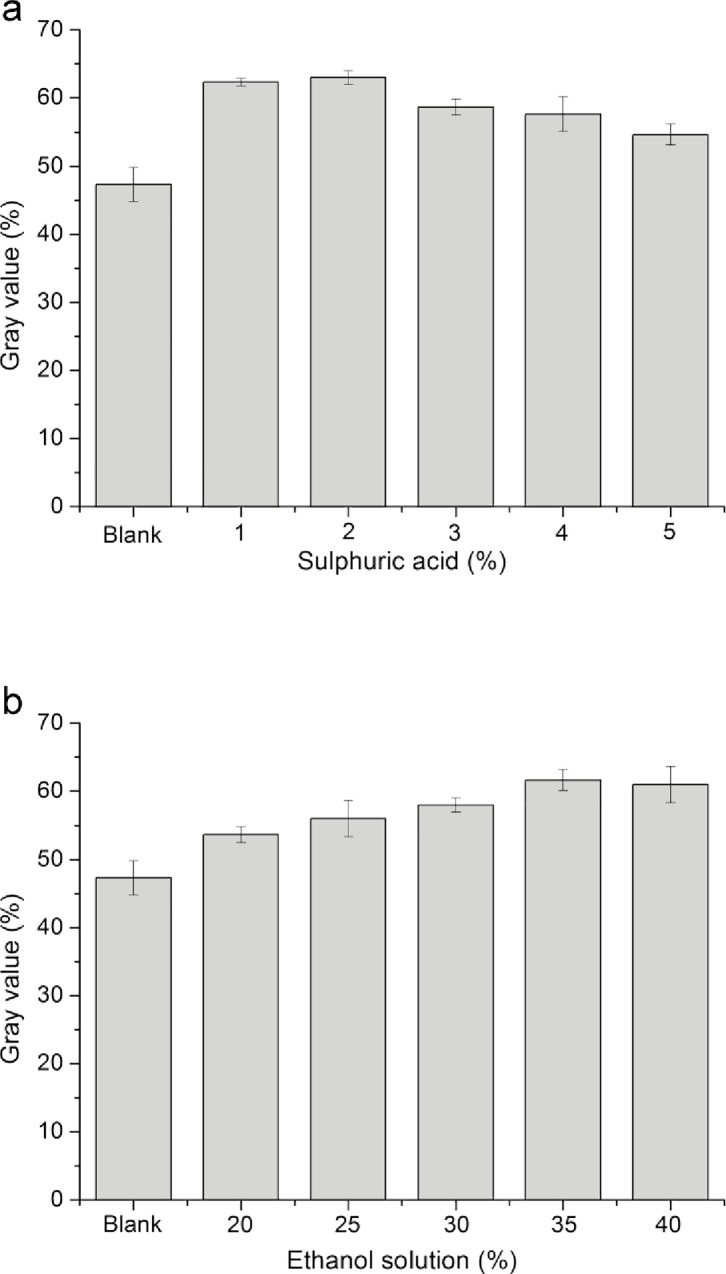
Effects of the amount of sulfuric acid and ethanol solution on the color assay. (a) Altered sulfuric acid percentage. (b) Altered ethanol solution percentage. Values are presented as means ± SD (n = 3).

The effects of the amount of sulfuric acid and ethanol solution on the color assay are displayed in Fig 4. The amount of sulfuric acid increased in the beginning, and the red precipitation formed more (Fig 4A). When the sulfuric acid percentage was 2%, namely 2 mL, the color displayed most obviously. However, as the percentage of sulfuric acid continued to rise, the red precipitation became less distinct. Afterward, the ethanol solution amount in the DNPH solution was regulated (Fig 4B). The red precipitation formed more with the increasing amount of ethanol solution. When the percentage of ethanol solution was 35%, which was 35 mL, its red precipitation produced the most across the reactions. Therefore, the optimal percentage of sulfuric acid in DNPH solution was 2% and that of ethanol solution was 35%.

### DNPH color assay application in the high-throughput screening mutants

This study applied cultivation mode of 24-deep-well plates to reduce the screening work period to achieve high-throughput screening. Thus, the growth status of the strain in 24-deep-well plates should be similar to or better than that of the corresponding shake flask; the optimal volume should be identified as well. Results are shown in Table 1, which indicates evaporation rate of the liquid and the strain’s growth at the 96th hour of the cultivation. A smaller volume was evident, and the liquid evaporation rate was higher. The liquid volume of 1.1 mL evaporated far faster than the others in the 24-deep-well plate and the shake flask of 20 mL culture. By contrast, the evaporation rate of 1.7 mL was closer to that of the shake flask, and the liquid tended to evaporate less than the shake flask when the volume was up to 2.0 mL. Almost all the volumes showed higher optical density values than the shake flask, which indicates that the strain can grow well or can even be better in 24-deep-well plates than in the shake flask. Combined with the consequence of the evaporation rate, the optimal liquid volume in 24-deep-well plates was chosen as 1.7 mL.

**Table 1:** Evaporation rate of the liquid and the strain’s growth in the medium of different volumes in 24-deep-well plates.

Volume (mL)	96^th^ hour	
	Evaporation rate (%)	Strain growth
20^a^	34.5±2.00	11.9±0.06	
1.1	64.2±6.39	11.3±0.32	
1.4	38.8±1.09	14.0±0.32	
1.7	35.7±5.30	13.9±0.36	
2.0	30.3±0.58	14.0±0.30	
2.3	27.0±0.87	14.2±0.55	

All experiments were carried out in triplicate.

^a^ indicates the volume of liquid in shake flask.

*R*. *rhodochrous* DSM43269 was treated with ARTP, and the resulting mutant colonies were inoculated, cultivated, and transformed AD in 24-deep-well plates under the optimal conditions obtained from the above work. The wild type was set as control to monitor substrate conversion. Transformation was terminated when the wild type totally decomposed AD. About 500 mutants were screened with DNPH, among which 59 mutants can form red precipitation. To ascertain that the steroids were 9α-OH-AD, their extracted organic phases were further assayed with TLC. As a result, only 5 mutants produced apparent 9α-OH-AD, whereas 54 samples either had a large amount of remaining AD or had other by-products.

**Fig 5:**
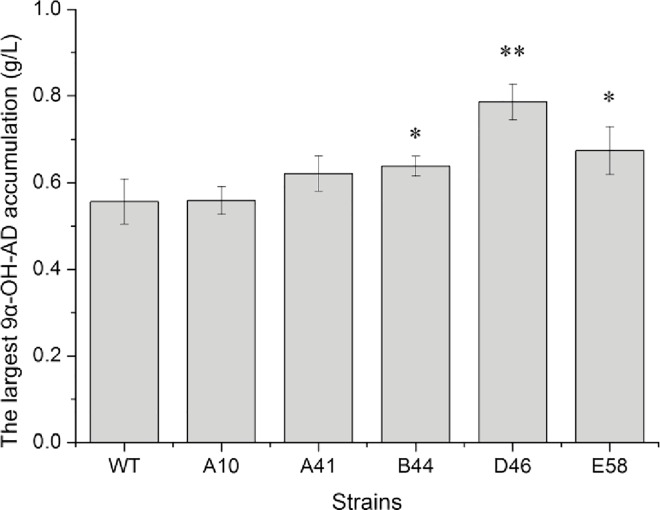
Transformation by shake flask and analysis of the largest 9α-OH-AD accumulation. The experiments were performed in triplicate. The standard deviations from three repeated experiments are shown. *t* test results are shown: *, P < 0.05; **, P < 0.01.

Afterward, these 5 mutants and the wild type were cultivated in the shake flask to precisely identify the accumulation of 9α-OH-AD, and the largest amounts of the product were compared. *R*. *rhodochrous* B44, D46 and E58 could accumulate more 9α-OH-AD than the wild type, especially D46, which remarkably produced 9α-OH-AD at about 0.2 g/L higher than the wild type (Fig 5).

## Discussion

During our daily research, we found the special character of this strain. In addition to 0.5 g/L of AD, we also inoculated the wild type *R*. *rhodochrous* DSM43269 in mineral medium with different AD concentration that ranged from 0 g/L to 3.0 g/L. Regardless of how much is the steroid concentration, the strain cannot normally grow in any steroid mineral medium that displays no significant difference from the control medium. On the contrary, much research work has reported that their strains can grow well on steroid mineral medium [9, 11, 12]. The possible reason is that KSDD and KSH activities of *R*. *rhodochrous* DSM43269 are weaker than those of the above strains used in the known literature [9, 11, 12]. *R*. *rhodochrous* DSM43269 cannot easily degrade AD via its KSDD and KSH to provide enough energy for its growth. By contrast, its system of metabolizing glucose is good so that it can take advantage of sufficient energy source to grow well. On the other hand, the colony morphology of *R*. *rhodochrous* DSM43269 appeared like a dry and rough plaster. Whenever the strain was inoculated, its colonies were found to easily shift to another place. Similarly, after the colonies on the gridded sterile membrane filters were dyed with DCPIP for 1 day, most fell off from the membranes. The special characters of *R*. *rhodochrous* DSM43269 make these two well-established methods impracticable to screen for its mutants that can accumulate 9α-OH-AD. *R*. *rhodochrous* DSM43269 can entirely degrade AD. The light absorption value of 254 nm wavelength of mutants that can accumulate 9α-OH-AD differed from that of the wild type. To achieve high-throughput screening, 96-well UV-transparent plates are necessary. However, these plates are very expensive, and the values obtained may be incorrect when reused for several times. Thus, the high-throughput screening method based on the principle that DNPH can react with aldehydes and ketones to form colored precipitation can competently substitute the light absorption value measurement. Moreover, numerous scientists have applied this principle in their detection and analysis work on aldehydes and ketones because of its simplicity and high sensitivity [13–15]. The method established in this work can rapidly distinguish mutants through the formation of red precipitation as long as 9α-OH-AD exists as they increase the efficiency of screening mutants. Various ketone compounds reacted with DNPH in our study, especially steroids with 3-keto-4-ene structure, all of which resulted in colored precipitation. Thus, this method is competent for screening the mutants of microorganism which can completely degrade a compound with no carbonyl group into one with a carbonyl group or vice versa, such as *Mycobacterium* transforming cholesterol into 9α-OH-AD not H_2_O and CO_2_. This simple and novel method will broaden screening methods and increase screening efficiency in microbial mutation breeding.
